# Implication of Ceramide Kinase/C1P in Cancer Development and Progression

**DOI:** 10.3390/cancers14010227

**Published:** 2022-01-04

**Authors:** Laura Camacho, Alberto Ouro, Ana Gomez-Larrauri, Arkaitz Carracedo, Antonio Gomez-Muñoz

**Affiliations:** 1 Department of Biochemistry and Molecular Biology, Faculty of Science and Technology, University of the Basque Country (UPV/EHU), 48080 Bilbao, Spain; laura.camacho@ehu.eus (L.C.); ana.gomezlarrauri@osakidetza.net (A.G.-L.); acarracedo@cicbiogune.es (A.C.); 2 Center for Cooperative Research in Biosciences (CIC bioGUNE), Basque Research and Technology Alliance (BRTA), Bizkaia Technology Park, Building 801A, 48160 Derio, Spain; 3 NeuroAging Group (NEURAL), Clinical Neurosciences Research Laboratories (LINCs), Health Research Institute of Santiago de Compostela (IDIS), 15706 Santiago de Compostela, Spain; alberto.ouro.villasante@sergas.es; 4 Respiratory Department, Cruces University Hospital, 48903 Barakaldo (Bizkaia), Spain; 5 IKERBASQUE, Basque Foundation for Science, 48009 Bilbao, Spain; 6 Centro de Investigación Biomédica en Red de Cáncer (CIBERONC), 28029 Madrid, Spain

**Keywords:** ceramide kinase, ceramide-1-phosphate, cancer cell signaling, tumor cell proliferation, invasion and dissemination

## Abstract

**Simple Summary:**

There are a number of reports in the scientific literature dealing with the implication of ceramide kinase (CERK) and its product, ceramide 1-phosphate (C1P), in the regulation of cell growth and survival, apoptosis, inflammation, and cell migration/invasion. However, no report has so far compiled or put into context the information related to the implication of the CERK/C1P axis in cancer development and metastasis. Hence, the present review highlights the relevance of CERK and C1P in tumorigenesis and tumor dissemination. Whilst CERK produces intracellular C1P, which can act on intracellular targets directly, C1P can also be secreted into the extracellular milieu and interact with sites (possibly receptors) at the plasma membrane of cells. This action can trigger signaling cascades that may end up modulating the expression of specific genes involved in tumor promotion and dissemination. The biology of CERK/C1P in cancer growth and dissemination is herein discussed in detail.

**Abstract:**

Cancer cells rewire their metabolic programs to favor biological processes that promote cell survival, proliferation, and dissemination. Among this relevant reprogramming, sphingolipid metabolism provides metabolites that can favor or oppose these hallmarks of cancer. The sphingolipid ceramide 1-phosphate (C1P) and the enzyme responsible for its biosynthesis, ceramide kinase (CERK), are well established regulators of cell growth and survival in normal, as well as malignant cells through stress-regulated signaling pathways. This metabolite also promotes cell survival, which has been associated with the feedback regulation of other antitumoral sphingolipids or second messengers. C1P also regulates cancer cell invasion and migration of different types of cancer, including lung, breast, pancreas, prostate, or leukemia cells. More recently, CERK and C1P have been implicated in the control of inflammatory responses. The present review provides an updated view on the important role of CERK/C1P in the regulation of cancer cell growth, survival, and dissemination.

## 1. Introduction

**Figure 1:**
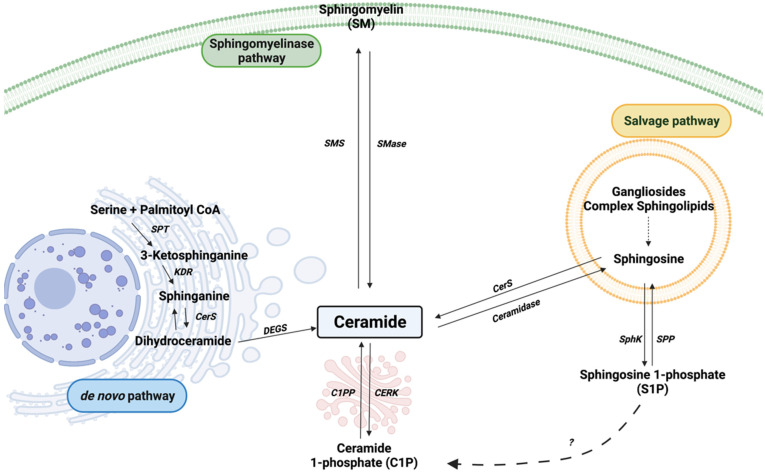
Sphingolipid metabolism. Sphingomyelinase (SMase), sphingomyelin synthase (SMS), serine palmitoyl transferase (SPT), 3-ketosphinganine reductase (KDR), ceramide synthase (CerS), dihydroceramide desaturase (DEGS), sphingosine kinase (SphK), sphingosine 1-phosphate phosphatase (SPP), ceramide 1-phosphate phosphatase (C1PP), and ceramide kinase (CERK) are represented by their acronyms. The dashed arrow with interrogation mark represents a putative mechanism for C1P generation from S1P.

Ceramide kinase (CERK) was first discovered in rat neural secretory (synaptic) vesicles by S. Bajjalieh and co-workers [1]. The enzyme activity was exclusively found in membrane fractions that contained synaptic vesicle markers, and its lipid product was ceramide-1-phosphate (C1P). Soon after, Kolesnick and Hemer [2] demonstrated the existence of C1P in human leukemia HL-60 cells and showed that C1P was mainly synthesized from sphingomyelin but not glycosphingolipids, although de novo synthesis of ceramides and the recycling of sphingosine in the salvage pathway are also substrates for CERK (Figure 1). Both the *de novo* synthesis and salvage pathways take place in the endoplasmic reticulum and are primarily regulated by ceramide synthases (CerS) [3,4].

**Figure 2:**
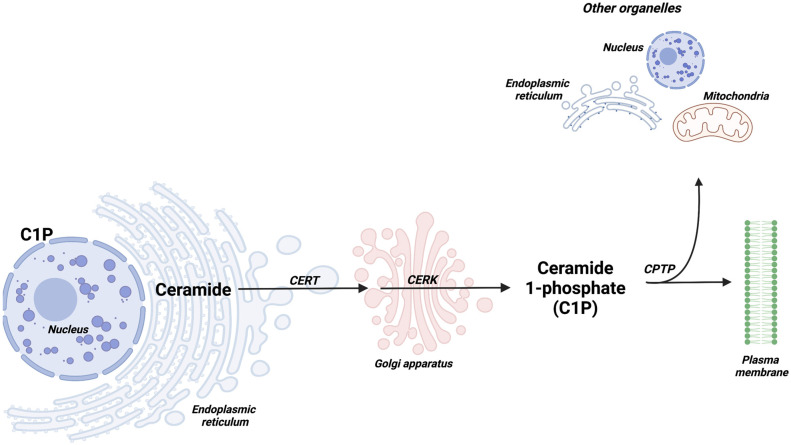
Synthesis and intracellular transport of C1P. Ceramides are transported from the endoplasmic reticulum (ER) to the Golgi apparatus by ceramide transfer protein (CERT). In the Golgi apparatus, ceramide kinase (CERK) phosphorylates ceramide to generate C1P. Then, a C1P transfer protein (CPTP) transports C1P to the plasma membrane and probably to other organelles. C1P is also present in the perinuclear region.

Once synthesized, ceramides can be transported by ceramide transfer protein (CERT) to the Golgi apparatus, where they can be phosphorylated by CERK to yield C1P (Figure 2). A specific ceramide phosphate transfer protein (CPTP) would then transport C1P to the plasma membrane and other organelles [5], where it may participate in the regulation of different cellular processes.

Besides CERK, the intracellular levels of C1P are regulated by hepatic ceramide phosphate phosphatase (CPP) [6] or lipid phosphate phosphatases (LPP) [7], enzymes that catalyze C1P dephosphorylation to increase ceramide levels. Non-conventional regulation of C1P production has also been reported. In this connection, sphingomyelinase phosphodiesterase-like 3b, an enzyme that is present in podocytes (terminally differentiated cells of the kidney filtration barrier), modulates C1P levels by preventing the access of CERK to its ceramide substrate [8], thereby reducing C1P levels.

Although C1P was discovered as early as in 1989, no biological function was attributed to CERK or C1P until 1995, with the discovery that C1P stimulated DNA synthesis and cell division in rat fibroblasts [9]. The mitogenic effects of C1P were accompanied by an increase in the levels of proliferating cell nuclear antigen (PCNA) [10] and were antagonized by (non-phosphorylated) ceramides [9,10]. Subsequent in vitro studies, using non-mammalian biological systems, showed that C1P increased the size of chick embryo otic vesicles, an action that was also accompanied by increased PCNA levels and inhibited by ceramides [11].

CERK and C1P regulate cell proliferation in a variety of cell types, including primary bone marrow-derived macrophages [12,13], mesenchymal cells [14], primary rat aortic vascular smooth muscle cells [15], primary photoreceptor progenitors [16,17], and renal mesangial cells and fibroblasts [18]. The mechanisms involved in the mitogenic actions of C1P in primary or transformed cells include the upregulation of mitogen-activated protein kinase kinase (MEK)/extracellularly regulated kinases 1-2 (ERK1-2), c-Jun N-terminal kinase (JNK), protein kinase Cα, NADPH oxidase, and phosphatidylinositol-3-kinase (PI3K)/Akt/mammalian target of rapamycin (mTOR) pathways [12,13,19,20,21]. Of interest, other molecular processes have been similarly involved in these phenotypes, including the C1P-mediated vascular endothelial growth factor (VEGF) production in macrophages [22] or C1P-induced lysophosphatidic acid (LPA) receptor activation in myoblasts [23]. In line with the latter report, Meacci and co-workers have recently shown that the CERK/C1P axis plays a crucial role as molecular regulator of skeletal muscle mass associated with cancer [24]. In addition to stimulating cell proliferation, C1P can increase cell number through the inhibition of apoptosis. The mechanisms by which C1P promoted mammalian cell survival involve the inhibition of the ceramide-generating enzymes acid sphingomyelinase (A-SMase) [25] or serine palmitoyl transferase (SPT) [26], stimulation of the PI3K/Akt pathway [27], and upregulation of the inducible form of nitric oxide synthase (iNOS) expression [28]. C1P was also found to be protective against cyclophosphamide-induced ovarian damage in a mouse model of premature ovarian failure [29], cisplatin ototoxicity in cochlear hair cells [30], TNFα-induced endothelial colony-forming cell apoptosis [31], and death of retina photoreceptors [16]. Of additional interest, inhibition of CERK was shown to block insulin-like growth factor-1-mediated survival of otic neurosensory progenitors by impairing Akt phosphorylation in chick embryos [32].

Another relevant biological function regulated by CERK and C1P is cell migration, a complex physiological process that is absolutely required for embryogenesis, organogenesis, wound healing, and immune responses. Whilst intracellularly generated C1P is implicated in the regulation of cell growth and survival, exogenous C1P is required for the regulation of cell migration in different cell types, including mouse and human monocytes/macrophages [33,34,35], human umbilical vein endothelial cells, multipotent stromal cells, and endothelial progenitor cells [36], stem cells in patients with acute myocardial infraction [37], coronary artery macrovascular endothelial cells [38], retina Muller glia cells [39], or bone marrow-derived mesenchymal stem cells [14]. Noteworthy, this biological function of C1P is also relevant in tumor cells. CERK-produced intracellular C1P has been shown to be required for invasion/migration of a variety of cancer cell types, as discussed below (reviewed in [40,41,42]). Two important molecular tools for studying the biology of CERK are the selective inhibitors NVP-231 [43,44] and K1 [45]. Although other inhibitors of sphingolipid metabolism may affect the levels of ceramide, which is the physiological substrate of CERK, these fall out of the scope of this review. Nonetheless, the reader is referred to a recent review by Gomez-Larrauri and co-workers to expand on these aspects [46]. Lastly, it should be noted that C1P is implicated in inflammatory responses [47,48] and CERK or C1P play key roles in adipogenesis [42,49,50]. All of the above cellular functions, namely the upregulation of cell proliferation, survival, and invasion/migration by CerK/C1P, are pro-tumorigenic actions and are hereby discussed in detail.

## 2. Implication of CERK/C1P in Leukemia Cell Growth and Dissemination

In addition to increasing proliferation of primary monocytes/macrophages (derived from the bone marrow), C1P turned out to be a potent stimulator of mouse leukemia RAW264.7 macrophage proliferation, an action that was dependent upon VEGF secretion from these cells [22]. C1P-stimulated VEGF release was substantially reduced by knocking down ERK2 or Akt2, without intervention of ERK1, Akt1, or Akt3, and was completely inhibited when PI3K was downregulated with specific siRNA, actions that led to complete inhibition of C1P-stimulated cell growth. Earlier studies, using the same leukemia cell model, showed that C1P stimulated cell migration. Interestingly, the role of this metabolite in regulating cell migration depends on its action on cell surface receptors rather than on its intracellular production and signaling. Neither the activation of CERK by interleukin-1β or the calcium ionophore A23187 [33] nor cell permeable light sensitive caged C1P analogs [35,51,52] could stimulate monocyte/macrophage migration suggesting that intracellular C1P is not essential for regulation of this process. However, treatment of the monocytes with C1P in the presence of pertussis toxin failed to stimulate ERK or Akt phosphorylation, as well as cell migration, suggesting the intervention of a Gi protein-coupled receptor in this action [33]. Similar results were obtained when human monocytic leukemia (THP-1) cells or J774.A1 reticulum cell sarcoma (a primary non-Hogdkin’s lymphoma of the bones) were used as biological models [34]. The latter report showed that C1P-stimulated cell migration was dependent upon ERK1-2 and Akt phosphorylation and the subsequent release of macrophage chemoattractant protein-1 (MCP-1). Moreover, C1P was present at relatively high levels in human leukemia (HL-60) cells, a hematological malignancy characterized by the accumulation of large numbers of immature myeloblasts in the bone marrow [2] and promoted homing of hematopoietic stem and progenitor cells that are susceptible to malignant transformation and development of leukemia [53]. Interestingly, prolonged incubation of human leukemia HL-60 cells, with the anticancer drug daunorubicin, rendered the cells resistant to this drug, a fact that was associated with reduced concentration of proapoptotic ceramides and a concomitant increase of antiapoptotic C1P levels [54]. Of additional interest, phosphatidic acid (PA), which is structurally related to C1P, displaced radiolabeled C1P from its membrane-binding site and inhibited C1P-stimulated mouse leukemia RAW264.7 cell migration. The mechanism by which PA exerted this inhibitory action was associated with a sharp reduction in the levels of phosphorylated ERK1-2. Exogenous bacterial phospholipase D (PLD) (from *Staphylococcus aureus*), an enzyme that produces PA at the plasma membrane of cells, recapitulated the inhibitory effects of exogenous PA on leukemia cell migration [35]. Since PA has mitogenic properties [55,56,57] and has been associated with progression of some cancer cell types [58,59,60,61,62], it might be possible that synthetic PA analogs lacking cell growth promoting properties could be useful for counteracting leukemia cell migration.

## 3. Implication of CERK/C1P in Breast Cancer

Breast cancer is the most common cancer among women worldwide, accounting for about 30% of female cancers, with a mortality-to-incidence rate of 15% [63]. Although primary tumors can be effectively treated by the combination of surgery, radiotherapy, and chemotherapy, many breast cancer patients relapse with recurrent disease, even 20 years after diagnosis and treatment, a fact that is associated with the morbidity and mortality of the disease [64]. Hence, identification of the mechanisms or pathways implicated in the survival of these cancer patients after therapy could contribute to the development of more efficient drugs or strategies to reduce the risk of recurrence. Analysis of gene expression profiles, from more than 2200 patients with breast cancer, revealed that *CERK* expression was associated with an increased risk of recurrence in women with breast cancer [64], suggesting that targeting this pro-survival enzyme may be highly beneficial to overcome this disease. Moreover, a recent report described the upregulation of CERK and sphingosine kinase 1, which produces sphingosine 1-phosphate (S1P), in breast cancer tissues. In particular the levels of C1P (23:0) and C1P (23:1) were elevated in tumor tissues, as compared to adjacent normal tissue, and correlated well with the Ki-67 index, a prognostic parameter in breast cancer patients [65]. Using the human breast cancer cell lines MDA-MB-231 and MCF7, Schwalm et al. showed that overexpression of CERK enhanced cell migration, through a mechanism involving activation of Akt [66]. The increased migration of CERK-overexpressing cells was reduced by pretreatment of the cells with the CERK inhibitor NVP-231 or by knocking down *CERK* expression with specific short hairpin RNAs. Moreover, inhibition of the PI3K/Akt pathway or RhoA-dependent protein kinase (ROCK) with the selective inhibitors LY290042 or Y27632, respectively, blocked migration of CERK-overexpressing breast cancer cells, suggesting a relevant role of these pathways in the regulation of CERK-associated breast cancer cell migration; targeting this enzyme may be an important therapeutic strategy in metastatic breast cancer. Of interest, it was also reported that estrogen receptor-negative breast cancer patients with high *CERK* expression exhibited worse prognosis [67]. Moreover, *CERK* overexpression was sufficient to promote triple-negative breast cancer (TNBC) cell growth and migration, and conferred chemoresistance to TNBC cell lines [68]. Moreover, inhibition of CERK counteracted chemoresistance in the breast cancer cells through a mechanism involving activation of the Ras/ERK, PI3K/Akt/mTOR, and RhoA pathways [68,69].

## 4. Implication of CERK/C1P in Lung Cancer

Lung cancer is the most devastating type of cancer worldwide. There are two major cancer subtypes, (1) non-small cell lung cancer (NSCLC), which is the most common lung cancer subtype, and accounts for 80–85% of all lung cancer cases [70], and (2) small cell lung cancer (SCLC), which is less frequent and accounts for 15–20% of the total lung cancer cases. The five-year lung cancer survival rate is approximately 18% [71]. Although surgery is the best choice for cure in early-stage NSCLC, many patients with lung tumors are not suitable surgical candidates because of limited pulmonary function and serious comorbidities [72]. Hence, in patients presenting metastatic disease, chemotherapy is the first-line treatment option [73]. Many anticancer drugs, including the most often used in lung cancer therapy (i.e., cisplatin, paclitaxel (taxol), gemcitabine, or etoposide), exert part of their anticancer activity by increasing the levels of proapoptotic ceramides [74,75,76,77,78]. Nonetheless, since the accumulation of ceramides might lead to C1P formation under conditions where CERK is upregulated or overexpressed, causing drug resistance, downregulation of this enzyme activity, or expression, would potentiate the chemotherapeutic actions of the anticancer drugs. In this concern, downregulation of CERK using specific siRNA reduced progression of the cell cycle into S phase, decreased cell proliferation, and enhanced apoptosis of the NSCLC A549 lung cancer cells [79]. Moreover, CERK inhibition using NVP-231 blocked breast and lung cancer cell proliferation by inducing M phase arrest and subsequent cell death [44]. Of particular interest is the fact that lung cancer is especially sensitive to the actions of extracellular C1P. Both lung cancer cell subtypes are sensitive to relatively low concentrations of C1P (0.5 µM) to undergo cell migration. Interestingly, C1P resulted to be more potent than its family member, S1P, at stimulating migration of the human lung cancer cells NSCLC A549, HTB177, HTB183, and CRL5803, as well as the SCLC CRL2062 and CRL5853 cancer cells [80]. Nonetheless, it has also been reported that contrary to the effects elicited by exogenous C1P on cell migration, intracellularly generated C1P (CERK-derived) inhibits cell migration and metastasis of the NSCLC A549 cells [81]. The latter report also shows that endogenous C1P inhibits the migration of MCF7 breast cancer cells. These C1P actions seem to be contradictory. However, and contrary to exogenous C1P, intracellular C1P accumulation failed to induce macrophage migration [35,52]. The latter findings could be recapitulated in a different study using 3T3-L1 cells to study preadipocyte differentiation into mature adipocytes. Specifically, it was observed that CERK expression increased during adipogenesis [49], whereas treatment of the preadipocytes with exogenous C1P inhibited this process [50]. Likely the effects of C1P may be dependent on different factors such as cellular compartmentalization, the ability of cells to secrete C1P into the extracellular milieu, or the different C1P species that can be produced at a time under specific metabolic conditions.

A malignancy associated with lung cancer is Kaposi sarcoma [82], which is a vascular tumor of the blood vessels and lymph nodes mainly linked to cutaneous lesions and is quite common in acquired immune deficiency syndrome (AIDS) patients [83]. Interestingly, endothelial colony-forming cells, a unique endothelial stem cell population, are highly increased in the blood of Kaposi sarcoma patients and have the ability to efficiently produce high levels of C1P (and S1P), a fact that significantly contributes to their increased proliferative properties. Additionally, exogenous C1P and S1P were found to stimulate proliferation of these cells [84], suggesting that both bioactive sphingolipids are relevant for development of Kaposi sarcoma; targeting C1P and S1P signaling may turn out to be a useful therapeutic approach to treat the disease.

## 5. Implication of CERK/C1P in Neuroblastoma

Our group previously reported that part of the mechanism whereby C1P stimulates primary bone marrow-derived macrophage proliferation involves activation of NADPH oxidase and the subsequent formation low levels of reactive oxygen species (ROS) [13], which contrary to the cytotoxic effects of high ROS concentrations, is a mitogenic signal [85,86,87,88,89]. C1P activated cPLA_2_ and PKCα in the macrophages and inhibition of these enzymes blocked C1P-stimulated NADPH activation, pointing to a relevant role of these kinases in the production of NADPH oxidase-derived ROS. Concurrently, Barth and co-workers showed that CERK regulates TNFα-stimulated NADPH oxidase and linked this action to the production of ROS and proinflammatory eicosanoids in human neuroblastoma cells [90]. Noteworthy, and in agreement with previous work, CERK was shown to be antiapoptotic and to suppress all-trans retinoic acid-induced neuronal differentiation in SH-SY5Y human neuroblastoma cells [91]. The proinflammatory actions of the CERK/C1P axis, along with its mitogenic and pro-survival properties, suggested a relevant role in the development of neuroblastoma, as well as in inflammation and the neuronal survival of the central nervous system. However, in normal mouse cerebellar Purkinje cells, CERK was not necessary for survival, although it played a relevant role in higher brain functions related to emotion [92]. Furthermore, in collaborative investigations with Meacci’s group, we found that the antiproliferative action of vitamin D_3_ and some of its synthetic analogues in human neuroblastoma cells implicated CERK. Specifically, the inhibition of this enzyme, by treatment with the active form of vitamin D_3_, 1,25-dihydroxivitamin D_3_, specific gene silencing, or with the pharmacological CERK inhibitor K1, led to a sharp depletion of intracellular C1P levels, and drastically reduced neuroblastoma cell proliferation [93]. The evidence that CERK/C1P acts as a molecular effector of the antiproliferative action of 1,25-dihydroxivitamin D_3_ represents a new possible target for anticancer therapy of human neuroblastoma and may help to identify new biomarkers for increased disease-specific risks in vitamin D_3_-deficient patients [93].

## 6. Implication of CERK/C1P in Pancreatic Cancer

Pancreatic cancer is the fourth leading cause of cancer mortality worldwide, with only 4–6% of patients with chances of a five-year survival rate after diagnosis [94,95]. Survival rates are better for patients with the malignant disease localized to the pancreas itself because surgical resection is practically the only chance of cure of this cancer type. However, 80–85% of patients with pancreatic cancer present with advanced unresectable disease, making it difficult to treat. Of interest, about 20% of pancreatic tumors are caused by cigarette smoke, and cancers from smokers show more genetic mutations than those from non-smokers [94]. The most common and deadly type of solid tumor in the pancreas is ductal adenocarcinoma, which has very poor prognosis. This aggressive disease is characterized by invasiveness, rapid progression, and profound resistance to chemotherapy. Hence, there is a need to better understand the biological mechanisms implicated in the establishment and progression of pancreatic tumors, so as to be able to develop effective interventions or better therapeutic strategies to treat this disease. C1P regulates cell migration and invasion of human pancreatic adenocarcinoma cells, actions that are necessary for cells to metastasize. Specifically, exogenous C1P enhances both migration and invasion of human ductal pancreatic PANC-1 and MiaPaCa-2 cells through a mechanism involving activation of the PI3K/Akt-1 and MEK/ERK1-2 pathways [96]. C1P also induces activation of the mammalian target of rapamycin (mTOR), which is downstream of Akt and upregulates the RhoA/ROCK-1 pathway, which is involved in reorganization of the cytoskeleton in the context of chemotaxis. Inhibitors of these and other signaling pathways are being used clinically to treat pancreatic cancer [97]. Interestingly, pretreatment of the pancreatic cancer cells with pertussis toxin, a Gi protein inhibitor, abrogated C1P-stimulated cell migration /invasion, suggesting the participation of a Gi protein-coupled receptor in these processes [96], as previously found in leukemia cells [33]. Moreover, pancreatic cancer cells have the ability to migrate spontaneously, and cells engineered to overexpress CERK showed enhanced spontaneous cell migration. The latter actions were completely blocked by NVP-231, or by downregulating CERK with specific siRNA in a receptor-independent manner [96]. Subsequent studies showed that pancreatic ductal adenocarcinoma cells secrete extracellular vesicles containing C1P to specifically promote pancreatic cancer stem cell motility, making C1P release a mechanism that could facilitate pancreatic tumor progression [98].

## 7. Implication of CERK/C1P in Prostate Cancer

Prostate cancer (PCa) is the second most frequent cancer diagnosed in men and fifth leading cause of death worldwide [99]. The androgen-signaling axis plays an essential role in the pathogenesis of prostate cancer [100]. The androgen receptor (AR) regulates multiple cellular events, such as proliferation, apoptosis, migration, invasion, and differentiation [101]. In turn, androgen receptors, as well as androgen synthesis, represent important therapeutic targets (androgen deprivation therapy), and a variety of anticancer drugs targeting this program have demonstrated to hinder prostate cancer progression [102]. However, resistance to androgen deprivation often arises, leading to castration resistant prostate cancer (CRPC), which accounts for a large fraction of prostate cancer mortality [103]. Studies focused on understanding the mechanisms underlying CRPC have increased the knowledge on dysregulated androgen signaling, leading to the development of several novel AR-directed therapies for CRPC (2).

In PCa, exogenous ceramide (C6) and (C16) treatment delayed the growth of tumor cells and induced cell death through apoptosis [104,105], thereby supporting the notion that agents that elevate ceramide levels could be used as novel chemotherapeutic agents [106].

Recently, our group has provided new insight into the regulation and function of CERK and C1P in PCa. Through a bioinformatics analysis of human PCa transcriptomics datasets, searching for metabolic genes correlated with AR activity, we found that CERK is among the top genes repressed by the nuclear receptor [107], thus confirming results obtained using other approaches [108]. We validated this regulatory mode using cell lines with AR agonists and antagonists and murine models of PCa subject to castration. Of importance, we showed that inhibition of AR, by means of MDV3100 treatment, increased the abundance of C1P (C20) and (C24). Molecular analyses confirmed that AR sits on CERK regulatory regions. Similar to the repression of NOV [109], the repressor EZH2 cooperates with AR to exert its regulation of CERK. Functional analyses confirmed that C1P treatment of PCa cells promoted cell migration (but not proliferation).

## 8. Concluding Remarks

**Figure 3:**
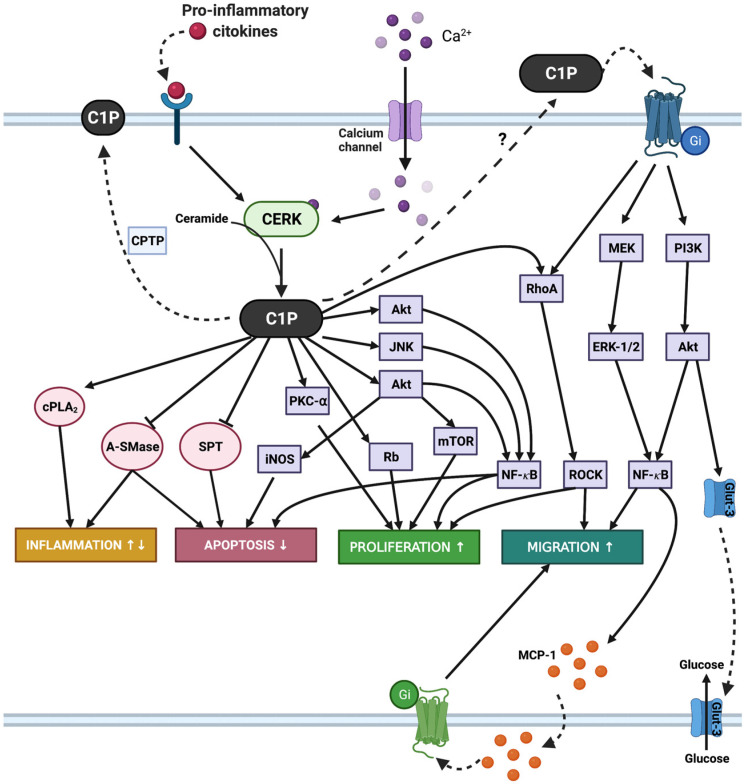
C1P biology in tumorigenesis. C1P is generated intracellularly by CERK, an enzyme dependent upon calcium ions for activity. C1P can be transported to the plasma membrane by CPTP or can be secreted into the extracellular environment. Intracellular C1P can exert various biological functions, including stimulation of cell growth, inhibition of apoptosis, stimulation of cell migration, or modulation of inflammation, for which it targets different signaling pathways. C1P can also act extracellularly to promote cell migration or glucose uptake, through interaction with a putative Gi protein-coupled receptor, also affecting various signaling pathways, as indicated.

It is well established that C1P and CERK regulate cell growth, survival, and motility in both malignant and non-malignant cells. Although the molecular mechanisms whereby CERK/C1P exerts its biological functions are still incomplete, accumulating evidence supports the notion that this enzyme activity and its product are relevant targets for reducing tumor formation and spreading. Taking into consideration the topology or compartmentalization of C1P in cancer cells, the therapeutic relevance of CERK and C1P can be pursued at two different levels. On one hand, as CERK is responsible for the generation of intracellular C1P, targeting this enzyme activity or expression is an exciting strategy to curb tumor growth. On the other hand, exogenous C1P, which is present in plasma and exosomes, can interact with putative Gi protein-coupled receptors to trigger cell migration/invasion in different cancer cell types. Although the putative C1P receptor(s) is (are) still to be fully characterized and cloned, these, together with CERK, may be highly relevant to promote the development of a new generation of cancer metabolism-targeting agents to aid in the prevention or cure of cancer. Figure 3 outlines the major signaling pathways that are regulated by C1P, in the context of tumor promotion, including cell growth and death, inflammation, and cell migration, which are all relevant aspects of tumorigenesis.
